# Supplementary material for: Neutralization and spike stability of JN.1-derived LB.1, KP.2.3, KP.3, and KP.3.1.1 subvariants
Source: mBio. 2025 Mar 26;16(5):e00464-25. doi: 10.1128/mbio.00464-25 (PMC12077133; doi:10.1128/mbio.00464-25)
Supplement: Supplemental Figures and Table — Table S1 and Fig. S1-S4. [file mbio.00464-25-s0001.pdf]

Table S1. Bivalent-vaccinated healthcare workers (HCWs) and BA.2.86/JN.1-wave patients

	Bivalent Health Care Workers (n=10)	**BA.2.86/JN.1 Wave Patients (n=10)**
Age in Years at Sample **Collection [Median (Range)]**	**37 (27-46)**	**52 (34-81)**
**Gender** [n (% of Total)]		
Male	**5 (50%)**	**6 (60%)**
Female	**5 (50%)**	**4 (40%)**
**Sample Collection Window**	**Dec. 2022- Jan.2023**	**Nov. 2023-Aug. 2024**
**Vaccine status** [n (% of Total)]	**NA**	
1-dose Pfizer	**NA**	**1(10%)**
2-dose Moderna	**NA**	**2 (20%)**
3-dose Moderna	**NA**	**1 (10%)**
4-dose Moderna	**NA**	**1 (10%)**
1-dose Moderna +1-dose Pfizer bivalent	**NA**	**1 (10%)**
1-dose Pfizer +1-dose Pfizer bivalent	**NA**	**2 (20%)**
2-dose Pfizer +1-dose Pfizer bivalent	**1 (10%)**	**NA**
3-dose Pfizer +1-dose Moderna bivalent	**NA**	**1 (14.3%)**
3-dose Pfizer +1-dose Pfizer bivalent	**3 (30%)**	**NA**
3-dose Pfizer +1-dose Moderna	**1 (10%)**	**NA**
3-dose Moderna +1-dose Moderna bivalent	**4 (40%)**	**1 (14.3%)**
2-dose Moderna +1 Pfizer +1-dose Pfizer bivalent	**1 (10%)**	**NA**
Days from last vaccination	**NA**	**675 (34-1033)**
Days post the bivalent dose for recipients	**66 (23-108)**	**NA**
**COVID-19 positive** [n (% of Total)]	**8 (80%)**	**10 (100%)**
Days before sample collection [(Median Range)]	**276.5 (182-994)**	**7 (1-10)**
**Infected Variants**		
JN.1/BA.2.86	**NA**	**2 (20%)**
Undetermined	**NA**	**8 (80%)**

Summary of the demographic information for two cohorts used for neutralization experiments depicted in Figure 2. “NA” means the category is not applicable to the cohort.

Supplementary figures and legends

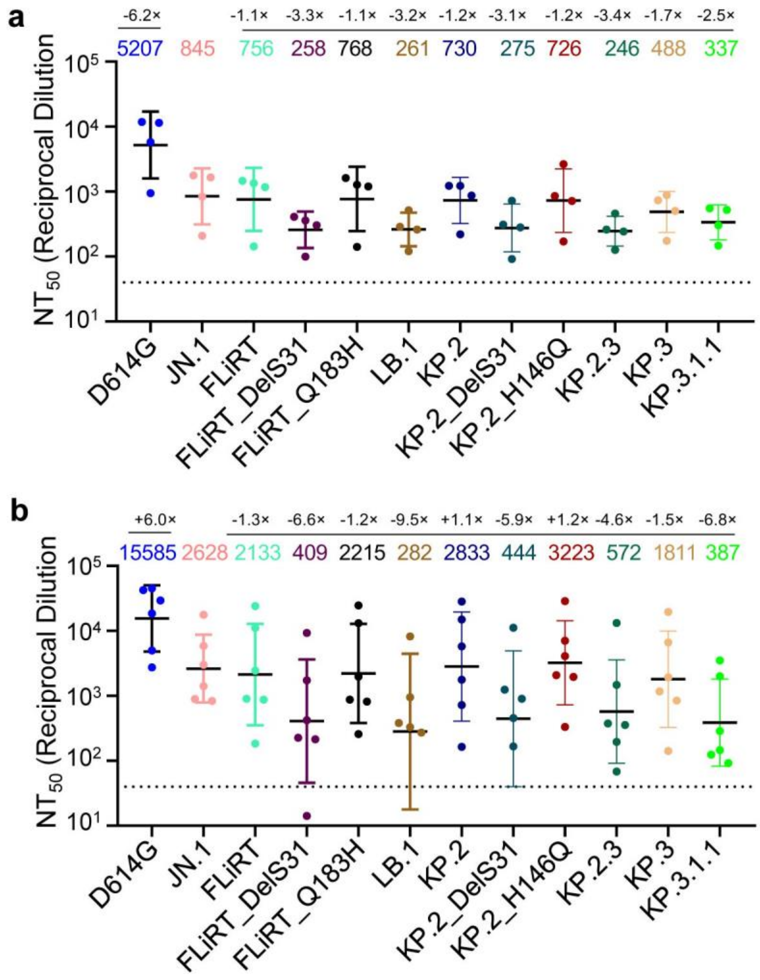

FIG S1. NAb titers in the sera of different cohorts. (a) First responders and household contacts during the BA.2.86/JN.1 in Columbus who became COVID positive and suffered mild illness (n = 4). (b) ICU patients during the BA.2.86/JN.1-wave in Columbus, Ohio (n=6).

Variants	IC_{50} (µg/ml)
D614G	0.15 ± 0.07
JN.1	> 12
FLiRT	> 12
FLiRT_DelS31	> 12
FLiRT_Q183H	> 12
LB.1	> 12
KP.2	> 12
KP.2_DelS31	> 12
KP.2_H146Q	> 12
KP.2.3	> 12
KP.3	> 12
KP.3.1.1	> 12

FIG S2. Neutralization of JN.1 variants by monoclonal antibody S309. Neutralization by class 3 monoclonal antibody S309 was determined for JN.1-derived variants of interest inhibitory concentrations at 50% (IC_{50}) was determined and displayed in (a). Raw luminescence values were normalized to untreated controls for plotting and IC_{50} calculations.

A

Bivalent HCWs			
AD (D614G)		AD (JN.1)	
JN.1	3.2	D614G	3.2
FLiRT	4.2	FLiRT	1.0
FLiRT_DelS31	6.4	FLiRT_DelS31	5.4
FLiRT_Q183H	3.9	FLiRT_Q183H	0.7
LB.1	6.4	LB.1	6.0
KP.2	3.7	KP.2	1.9
KP.2_DelS31	6.1	KP.2_DelS31	4.7
KP.2_H146Q	3.9	KP.2_H146Q	2.4
KP.2.3	6.4	KP.2.3	5.6
KP.3	4.0	KP.3	1.6
KP.3.1.1	6.5	KP.3.1.1	5.3

B

BA.2.86/JN.1-wave patients			
AD (D614G)		AD (JN.1)	
JN.1	2.3	D614G	2.3
FLiRT	2.7	FLiRT	1.0
FLiRT_DelS31	5.0	FLiRT_DelS31	4.0
FLiRT_Q183H	2.7	FLiRT_Q183H	1.1
LB.1	5.3	LB.1	4.5
KP.2	2.4	KP.2	0.9
KP.2_DelS31	4.8	KP.2_DelS31	3.9
KP.2_H146Q	2.4	KP.2_H146Q	0.8
KP.2.3	4.7	KP.2.3	3.0
KP.3	3.2	KP.3	1.5
KP.3.1.1	4.8	KP.3.1.1	4.6

C

XBB.1.5-monovalent hamsters			
AD (D614G)		AD (JN.1)	
JN.1	1.7	D614G	1.6
FLiRT	1.8	FLiRT	0.4
FLiRT_DelS31	2.9	FLiRT_DelS31	1.9
FLiRT_Q183H	1.6	FLiRT_Q183H	0.4
LB.1	2.8	LB.1	1.8
KP.2	1.9	KP.2	0.3
KP.2_DelS31	2.8	KP.2_DelS31	1.5
KP.2_H146Q	2.1	KP.2_H146Q	0.5
KP.2.3	3.0	KP.2.3	1.8
KP.3	1.9	KP.3	0.3
KP.3.1.1	2.8	KP.3.1.1	1.5

FIG S3. Antigenic distances of JN.1-derived subvariants relative to D614G or JN.1 in three groups of cohorts. (a) bivalent-vaccinated HCWs, (b) BA.2.86/JN.1-wave infected people, and (c) XBB.1.5-vaccinated hamsters. One antigenic distance unit (AU) is equivalent to a 2-fold difference in nAb titer shown in Figure 2.

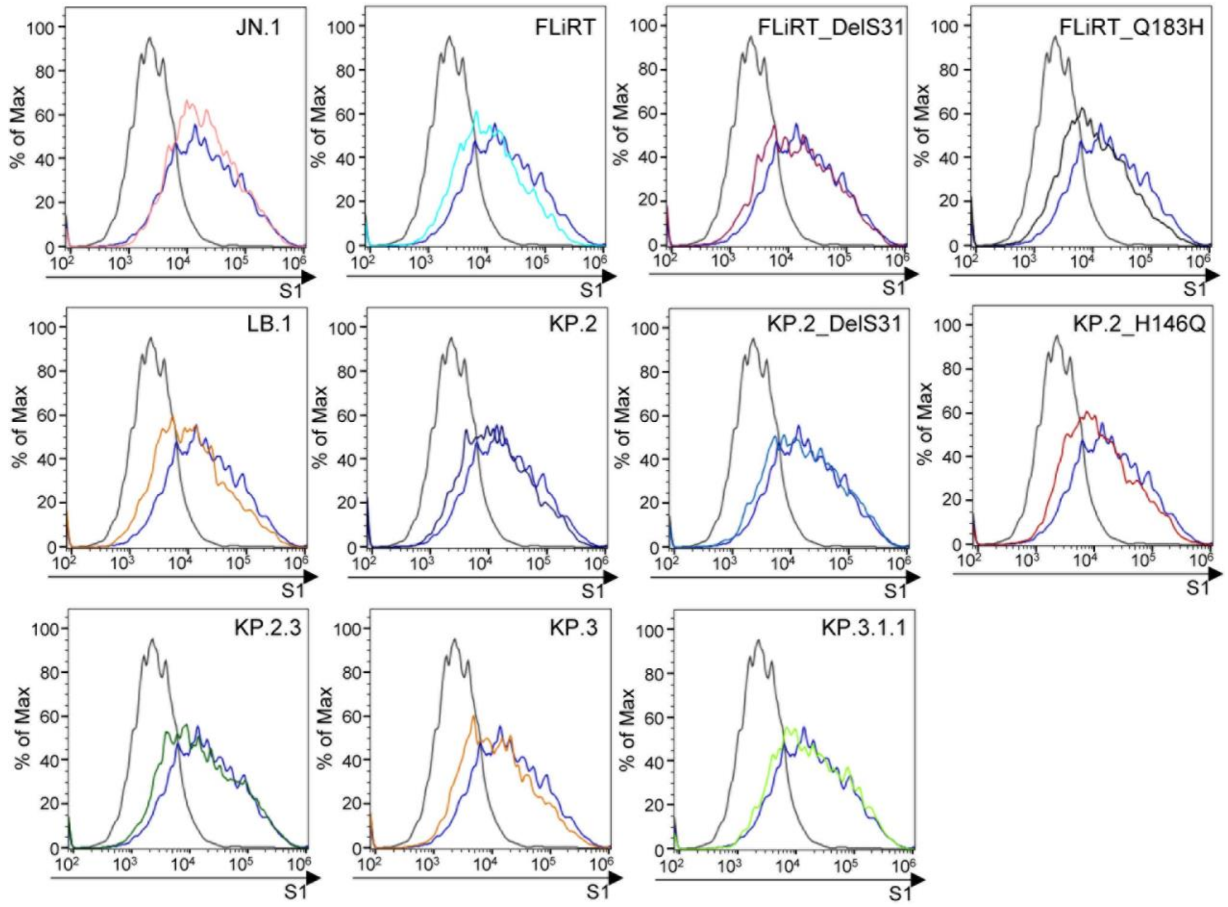

FIG S4. Expression and processing of JN.1-derived spike protein on the plasma membrane compared to D614G. 293T cells used to produce pseudotyped vectors was probed with anti-S1 antibody to compare surface expression between spikes of interest. The gray lines represent "No-Spike", the blue lines represent "D614G", and the other colored lines correspond to the Spike "variant of interest" as indicated.
